# Rotating Night Shift Work, Exposure to Light at Night, and Glomerular Filtration Rate: Baseline Results from a Chinese Occupational Cohort

**DOI:** 10.3390/ijerph17239035

**Published:** 2020-12-04

**Authors:** Shengkui Zhang, Yongbin Wang, Ying Zhu, Xiaoming Li, Yang Song, Juxiang Yuan

**Affiliations:** 1 Department of Epidemiology and Health Statistics, School of Public Health, North China University of Science and Technology, Tangshan 063210, China; zhangsk@stu.ncst.edu.cn (S.Z.); zhuying@ncst.edu.cn (Y.Z.); lixiaoming@ncst.edu.cn (X.L.); songyang@ncst.edu.cn (Y.S.); 2 Department of Epidemiology and Health Statistics, School of Public Health, Xinxiang Medical University, Xinxiang 453003, China; 191035@xxmu.edu.cn

**Keywords:** renal function, light at night, night shift work

## Abstract

The misalignment between the circadian clock and behavioral cycles has been implicated in pathogenesis of many diseases. The main purpose of this study is to examine the association between rotating night shift work, exposure to light at night, and glomerular filtration rate among steelworkers in north China. A total of 6869 steelworkers, aged 22 to 60 years, were included in this study. Multivariable logistic regression was used to examine the association between night shift work, the brightness of bedroom ambient light at night (LAN), and estimated glomerular filtration rate (eGFR), with adjustment for potential confounders. Mediation analysis was performed to examine the mediation effect of potential mediators on the association of duration of night shifts and eGFR. Long duration of night shift work (≥29 years) had elevated odds of decreased eGFR (≤89 mL/min/1.73 m^2^) (odds ratio (OR), 1.37, 95% confidence interval (CI) 1.09–1.73) compared with day work after adjustment for potential confounders. The association between duration of night shifts and eGFR (continuous) was partially modified by diastolic blood pressure (average causal mediation effect (ACME), –0.077, 95% CI –0.134 to −0.030, *p* < 0.001). No significant associations were observed among the different brightness of bedroom ambient light levels: middle level (OR, 0.90, 95% CI 0.77–1.05), lightest level (OR, 0.94, 95% CI 0.75–1.18), and decreased eGFR compared with the darkest level. Long-term night-shift work, rather than the brightness of bedroom ambient LAN, is associated with early stage of renal dysfunction in steelworkers, and blood pressure may mediate the relationship between night shift work and decreased eGFR.

## 1. Introduction

To maximize economic and societal benefit, modern society is dependent on a 24 h schedule. In this circumstance, rotating night shift work and exposure to artificial light at night (ALAN) have become commonplace, even though its negative impact on health has been shown in a considerable body of evidence from epidemiological and experimental studies [1]. Approximately 20% of workers in industry countries are engaged in a shift work schedule [2]. In addition to the night shift work, which inevitably exposes workers to LAN during their working hours, ALAN in the daily life has become a widespread environmental pollutant. It is estimated that 23% of the land surface experienced ALAN [3], and this exposure is increasing about 6% per year [4]. Moreover, the trend of exposure to night shift work and LAN parallels the increase in the percentage of patients with chronic kidney disease (CKD) in China [5].

The overall prevalence of CKD in China was 11.6% [6], and the spectrum of CKD in China has been evolving toward that of developed countries [5]. Identification of the risk factors for early stage of renal dysfunction, such as mildly decreased glomerular filtration rate (GFR), is essential for reducing the burden of CKD. In fact, several renal functions, including glomerular filtration rate, have circadian rhythms [7]. Since the mid-19th century, circadian rhythms of most renal functions have been published [7]. The discovery of its molecular mechanism, the circadian clock, which has been recognized with the awarding of the Nobel Prize in Physiology and Medicine in 2017, has brought the study of renal circadian and its health consequences to a new era [8]. Misalignment between behavioral and molecular circadian clocks due to night shift work may increase the risk of sleep disturbances [9], which has been implicated in pathogenesis of CKD [10]. In addition, shift-workers are subject to heavier stress loads and are inevitably exposed to LAN [11]. Circadian rhythm disruption by shift work or LAN may prompt over-activation of the renin-angiotensin-aldosterone system or the sympathetic nervous system, which are known risk factors of decreased renal function [12]. Moreover, many analyses have shown that shift work may be associated with kidney damage and even chronic kidney disease, regardless of the duration of night shifts [13,14,15,16]. However, it is unclear whether the chronic disruption of circadian rhythms due to LAN or night shift work is responsible for the early stage of chronic kidney disease. To the best of our knowledge, there is currently no other study to examine the relationship between rotating night shift work, exposure to LAN, and mildly decreased estimated glomerular filtration rate (eGFR) based on a large-scale population. The aim of this study is to explore whether rotating night shift work and exposure to bedroom ambient LAN are associated with the early stage of renal dysfunction.

## 2. Materials and Methods

### 2.1. Study Design and Population

This cross-sectional study reported results from the baseline survey conducted among steelworkers who were prospectively recruited at eleven steel production departments in north China [17]. All workers at this company underwent a legally required health examination each year. A total of 7661 participants were recruited from February to June 2017. After excluding 390 participants without detailed information on current shift work status, 104 without brightness of bedroom ambient LAN, 43 without serum creatinine, and 255 with incomplete information on covariates, 6869 participants were ultimately included in this cross-sectional study (Supplementary Figure S1). Compared with the included participants, those who were excluded from this analysis had a higher proportion of male workers (93.7% versus 91.5%, *p* = 0.032) and were older (44.2 ± 8.0 years versus 34.3 ± 7.6 years, *p* < 0.001). This research was approved by the Ethics Committee of North China University of Science and Technology (No.16040). All participants gave informed consent before taking part in this study.

### 2.2. Assessment of GFR

Blood was drawn from the participants’ forearm venous between 08:00 and 09:30 after they have fasted for 12 h. For night shift workers, blood was drawn in the morning after a day off work. The sarcosine oxidase method was used to test serum creatinine (CRE kit, Beijing Strong Biotechnologies, Inc., Beijing, China). Within-laboratory intra- and inter-assay variable coefficients for serum creatinine were <6% and <8%, respectively. Assessment of eGFR was based on the Chronic Kidney Disease Epidemiology Collaboration (CKD-EPI) equation [18]. The CKD-EPI formula is as follows: eGFR (mL/min/1.73 m^2^) = 141 × min (Scr/κ, 1) ^α^ × max (Scr/κ, 1) ^−1.209^ × 0.993 ^Age^ × (1.018 if female) × (1.159 if black). Scr indicates serum creatinine (μmol/L), κ = 0.7 for females and 0.9 for males, α = −0.329 for females and −0.411 for males, min and max indicate the minimum of Scr/κ or 1, the maximum of Scr/κ or 1, respectively. According to the Kidney Disease Improving Global Outcomes 2012 recommendations, the range of GFR (mL/min/1.73 m^2^) was classed into five categories: normal or high (GFR ≥ 90, G1), mildly decreased (GFR: 60–89, G2), mildly to moderately decreased (GFR: 45–59, G3a), moderately to severely decreased (GFR: 30–44, G3b), severely decreased (GFR: 15–29, G4), and kidney failure (GFR < 15, G5) [19]. Based on these classification criteria, only 22 (0.3%) participants had eGFR < 60 mL/min/1.73 m^2^ (G3a–G5), so we combined these categories into G2 in the subsequent analysis and defined them as “decreased eGFR”.

### 2.3. Assessment of Shift Work

The main work schedule of the present study population has been introduced in detail in our previous research [17]. In brief, shift work in this study refers to rotating night shifts (mainly the four-crew-three-shift system used now and the historical three-crew-two-shift system). Workers who worked regular working hours at all times were defined as day workers. In this study, the detailed lifetime employment history was collected by face-to-face personal interviews. Participants recruited were asked to report whether they were involved in rotating night shift work (working through 00:00 to 6:00) during their employment [20]. If yes, they would be further asked about the start and end dates of each shift system, the average number of night shifts per month in each shift system, and usual days off per month. All the reported information was verified with the company’s records. “Day work” indicates workers who always worked regular working hours (8:00 to 16:00). Using the above work schedule information, the duration of night shift work (years) (sum of years spent in all different night shift systems) and cumulative number of night shifts (nights) (sum of nights spent in all different night shift systems) were aggregated.

### 2.4. Assessment of Bedroom Light Environment

Exposure to LAN was assessed through participants’ reports about the brightness of the bedroom ambient at night. Participants were asked to class the brightness of their bedroom LAN into the following four categories: “you wear a mask or too dark to see your fingers”, “light enough to see your fingers but not to identify the indoor environment clearly”, “light enough to identify the indoor environment clearly but not enough to read”, and “light enough to read”. The two lightest categories were combined because of small numbers in the lightest category (2.01%). Finally, brightness of the bedroom ambient at night was divided into three categories: “darkest”, “middle”, “lightest”. The participants were also asked to report the usual number of times per night the subject’s sleep was interrupted due to light and living duration of current residence [21,22].

### 2.5. Assessment of Covariates

Covariates mainly included age, sex, body mass index (BMI), ethnicity, smoking status, drinking status, educational level, physical activity, sleep duration, insomnia, diabetes, hyperuricemia, and hypertension (see Supplementary Materials).

### 2.6. Statistical Analysis

Continuous variables are presented as means and standard deviations, and between-group comparisons were performed using Student’s t-test if the data were normally distributed. Otherwise, the median (upper quartile–lower quartile) and Wilcoxon Scores (Rank Sums) test were used to describe and compare these continuous variables between groups. Initial bivariate associations between eGFR and potential confounders were also assessed with Pearson’s correlation (*r*) or Spearman’s rank correlation (*r_s_*) for continuous variables according to its distribution characteristics. Categorical variables are presented as numbers and percentages, and the chi-square test was used to compare differences among groups.

Multivariate logistic regression models were used to examine the relationships between night shift work, the brightness of bedroom ambient, and decreased eGFR. The risk factors of CKD reported in the literature (age, sex, diabetes, hypertension, obesity, hyperuricemia, short sleep duration, sleep quality, and socioeconomic status) [5,10,19], the potential confounders with unbalanced distribution between shift and day workers, and the factors influencing the assessment of LAN exposure in life were included in the multivariate analysis.

Restricted cubic spline models were utilized to visually examine the association between duration of night shifts (continuous), cumulative number of night shifts (continuous), and eGFR (as continuous and categorical variables, respectively), with adjustment for potential confounders.

Subsequently, in subgroup analysis, we introduced multiplicative interaction terms using the duration of night shifts in quartiles and the stratifying factors including sex, BMI (<25, or ≥25 kg/m^2^), bedroom ambient light level (darkest level/middle or lightest level), diabetes (no/yes), hypertension (no/yes), hyperuricemia (no/yes), insomnia (no/yes), and short sleep duration (no/yes) to assess potential effects modification. Log likelihood ratio test was used to compare models with and without cross-product interaction terms. Potential mediators of the association between duration of night shifts (ordered variable: 0, day work; 1, 1–12 years; 2, 13–20 years; 3, 21–28 years; 4, 29–43 years) and eGFR (continuous variable), including systolic blood pressure (continuous variable), diastolic blood pressure (continuous variable), fasting blood glucose (continuous variable), serum uric acid (continuous variable), sleep duration (continuous variable), and Athens Insomnia Scale (AIS) score (continuous variable), were introduced to estimate the average causal mediation effects (ACME) using a R package “mediation” (version 4.5.0) [23]. *p* < 0.05 was regarded as significant for 2-sided tests.

## 3. Results

### 3.1. General Characteristics of the Participants

The baseline characteristics according to the eGFR status are shown in Table 1. The present study of 6869 participants consisted of 91.5% males, with a mean age of 44.2 years, a mean BMI of 25.2 kg/m^2^, and a mean eGFR of 101.7 mL/min/1.73 m^2^. Approximately 85% of participants currently or previously engaged in night shift work. 11.8% of participants reported a lightest brightness of bedroom ambient LAN exposure. 25.6% of participants reported hypertension and 10.5% diabetes. The prevalence of decreased eGFR in day workers (0 years), 1–12 years (Q1), 13–20 years (Q2), 21–28 years (Q3), and 29–43 years (Q4) of night shifts in night shift workers were 13.2%, 15.3%, 16.6%, 21.0%, and 34.0%, respectively. Current smoking and current drinking were more likely to be reported among night shift-workers (Supplementary Table S1). Compared with female workers, male workers had higher proportions of smoking, drinking, diabetes, hypertension, and hyperuricemia (Supplementary Table S2). eGFR was correlated with BMI (*r* = −0.053, *p* < 0.001), sleep duration (*r* = 0.063, *p* < 0.001), fasting blood glucose (*r_s_*= −0.123, *p* < 0.001), systolic blood pressure (*r* = −0.136, *p* < 0.001), diastolic blood pressure (*r* = −0.167, *p* < 0.001), and duration of night shift work (*r* = −0.201, *p* < 0.001).

### 3.2. Night Shift Work, LAN and eGFR

**Figure 1:**
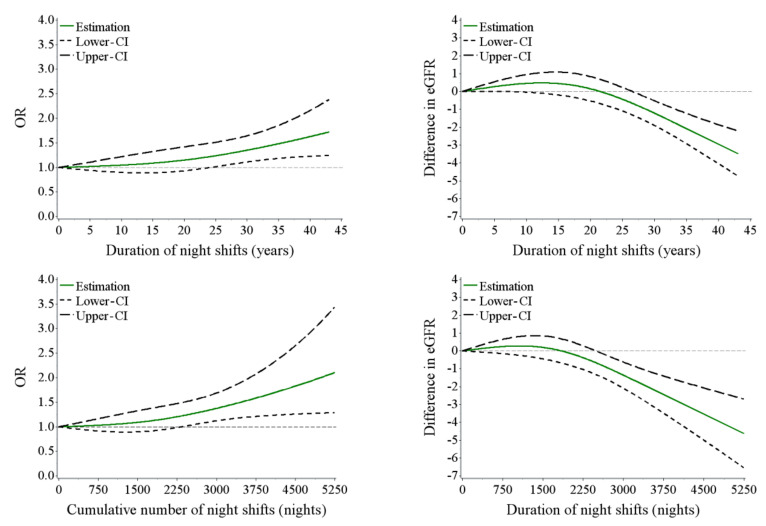
Associations of duration of night shifts (continuous), and cumulative number of night shifts (continuous) with eGFR. “Difference in eGFR” indicates difference in eGFR (mL/min/1.73 m^2^), where the reference values for duration of night shifts and cumulative number of night shifts are all 0 (day work). Adjusted for age, sex, BMI (<25, 25–30, or ≥30 kg/m2), smoking status, drinking status, education level, short sleep duration (<7 or ≥7 h), living duration of current residence, number of times light on, insomnia, diabetes, hypertension, and hyperuricemia. BMI, body mass index; eGFR, estimated glomerular filtration rate; OR, odds ratio; CI, 95% confidence interval.

The independent effect of night shift work and bedroom ambient LAN level on odds of decreased eGFR are shown in Table 2. Compared with day work, significantly increased odds ratios (OR) of decreased eGFR were observed in the higher exposure groups of “duration of night shift work (years)” among night shift workers (OR = 1.29, 95% confidence interval (CI): 1.02 to 1.63, in Q3; OR = 1.89, 95% CI: 1.52 to 2.36, in Q4; *p* trend < 0.001, Model 1). After adjustment for age and sex (Model 2), this association remained robust (OR = 1.32, 95% CI: 1.04 to 1.68, in Q3; OR = 1.40, 95% CI: 1.12 to 1.75, in Q4; *p* trend = 0.001, Model 1). After additionally adjusting for BMI, smoking status, drinking status, education level, short sleep duration, living duration of current residence, number of times light on, insomnia, diabetes, hypertension, and hyperuricemia, the odds of decreased eGFR in the last quartile (Q4) of the duration of night shifts was attenuated but remained significantly elevated with the OR (95% CI) of 1.37 (1.09 to 1.73) (Model 3). In addition, positive associations were observed between duration of night shifts (continuous, years), cumulative number of night shifts (continuous, nights), and odds of decreased eGFR (bivariate, yes/no), while negative associations were observed between duration of night shifts (continuous, years), cumulative number of night shifts (continuous, nights), and eGFR (continuous, mL/min/1.73 m^2^) in the restricted cubic splines (RCS) models (Figure 1). However, no significant association between brightness of bedroom ambient LAN and decreased eGFR was observed, regardless of whether the potential confounders were adjusted (Table 2). In addition to the bedroom ambient LAN, night shift workers were also inevitably exposed to LAN during their working hours. To minimize residual confounding, we changed the night shift exposure metric to cumulative number of night shifts (quartiles) instead of the duration of night shifts. After mutually adjusting for the cumulative number of night shifts, BMI, smoking status, drinking status, education level, sleep duration, living duration of current residence, number of times light on, insomnia, diabetes, hypertension, and hyperuricemia, no significant association between brightness of bedroom ambient LAN and odds of decreased eGFR was observed, with the ORs (95% CI) in middle and lightest group of 0.90 (0.77 to 1.06) and 0.95 (0.76 to 1.19), respectively (Supplementary Table S3).

### 3.3. Sensitivity Analyses

We analyzed the relationship between duration of night shifts and decreased eGFR through stratification analysis based on potential effect modifiers (Table 3). Compared with day work, elevated odds of decreased eGFR were observed in the higher exposure groups of the duration of night shifts in all subgroup analyses, except for the female group, although this association was no longer significant in some subgroups due to case numbers. There was no significant effect modification of the association between duration of night shifts and decreased eGFR by sex, BMI, brightness of bedroom ambient LAN, diabetes, hypertension, hyperuricemia, insomnia, and short sleep duration (all *p* for interaction > 0.05).

It is well-known that CKD has been linked to diabetes, hypertension, hyperuricemia, short sleep duration, and sleep disturbances, which also potentially could be direct consequences of exposure to shift work, thus making these factors potential mediators of the association between shift work and eGFR. After adjustment for age, sex, BMI, smoking status, drinking status, education level, short sleep duration, living duration of current residence, number of times light on, insomnia, diabetes, and hyperuricemia, the ACME of diastolic blood pressure (continuous) on the association of duration of night shifts (ordered variable) and eGFR (continuous variable) was −0.077 (95% CI (−0.134 to −0.030, *p* < 0.001)) (Table 4). The association between duration of night shifts (ordered variable) and eGFR (continuous variable) did not appear to be modified by systolic blood pressure (continuous variable), fasting blood glucose (continuous variable), serum uric acid (continuous variable), sleep duration (continuous variable), and AIS score (continuous variable, range: 0 to 24) (ACMEs crossed 0) (Supplementary Figure S2).

Considering that dust, heat stress, noise, and carbon monoxide are the major occupational hazards to the current steelworkers, we further adjusted these exposures on the basis of Model 3 in Table 2, and the results were similar to those in Table 2 (Supplementary Table S4). Moreover, in order to avoid the influence of the maximum value on the fitting results of restricted cubic splines, we removed the last 1% quantile of the duration of night shifts and cumulative number of night shifts, and the relationships remained comparable to Figure 1 (Supplementary Figure S3). Indeed, the medical history with current medications may affect blood pressure and therefore the decreased eGFR. In order to exclude the decrease in eGFR from medications and effect of family history of CKD, we further adjusted the previous history, medication status, and duration of diabetes, hypertension, and glomerulonephritis, as well as family history of CKD on the basis of Model 3 in Table 2, and the results were comparable to those in Table 2 (Supplementary Table S5). In addition, we also replaced the exposure metric of night shift work from the duration of night shifts to the cumulative number of night shifts (quartiles). After further adjustment for the previous history, medication status, and duration of diabetes, hypertension, and glomerulonephritis, as well as family history of CKD on the basis of Supplementary Table S3, the results remained robust (Supplementary Table S6).

## 4. Discussion

Our findings support that night shift work is significantly associated with early stage of renal dysfunction in steelworkers, and provide additional evidence concerning dose-response relationships between duration of night shifts, cumulative number of night shifts, and eGFR among night shift workers, which have never been reported in previous studies. However, no significant association is observed between the brightness of bedroom ambient LAN and decreased eGFR.

Our findings are consistent with a small sample cross-sectional study conducted in 354 police officers, which concluded that night shift work was associated with decreased kidney function [15]. They also reported that percentage of hours worked on the night shift work was inversely associated with mean levels of eGFR, which was comparable to our results when it comes to the relationship between duration of night shifts, cumulative number of night shifts, and eGFR. In line with our findings, another observational study also reported that small increase in albuminuria, a marker of kidney damage, was associated with disruption of the circadian rhythms due to shift work rather than exposure to low concentrations of nephrotoxic chemicals [16]. In addition, data from the Korea National Health and Nutrition Examination Survey also showed that shift work was associated with microalbuminuria and the risk of CKD in female workers [13,14]. Notably, the association of night shift work and decreased eGFR was limited to male workers in our study. This may be related to the protective effects of estrogens in women and/or the damaging effects of testosterone, together with unhealthier lifestyles in men, which might cause renal function to decline faster in men than in women [24]. Taking into account the small sample size of female workers in our study, the relationship between night shift work and decreased eGFR in females needs to be further studied.

What we already know is that the central oscillator for circadian rhythms in mammals resides in the suprachiasmatic nucleus (SCN) of the hypothalamus, and light serves as the primary “zeitgeber”. Asynchrony between circadian clocks and external world light/dark cycle due to LAN has been associated with increased urine production of the kidney [25]. However, no relationship was discovered between the brightness of bedroom ambient LAN and decreased eGFR in our study. This may be related to the overall lower exposure level of bedroom ambient LAN in our study population. In addition, considering that approximately 85% of participants are current or previous night shift workers, which is also a main cause of exposure to LAN, therefore, the absence of brightness assessment during night work may result in bias. But this bias could be largely controlled after adjustment for the cumulative number of night shifts, since night shift workers in this company spend most of their working hours in the central control room where the indoor environment was built according to uniform standards with the unified lighting system.

Interestingly, a partial mediation effect of diastolic blood pressure on the relationship between duration of night shifts and eGFR was observed in this study. It is well-known that hypertension could be a direct consequence of exposure to rotating shift work [26]. In addition, previous evidence showed that both central blood pressure and peripheral blood pressure were independently associated with mildly decreased GFR [27]. Moreover, a large population-based study from China (33,300 Chinese adults) showed that hypertension was significantly associated with mildly, moderately, and even severely decreased eGFR risk [28]. It is possible that hypertension might alter renal hemodynamics by increasing peripheral resistance and/or blood flow pulsation, leading to renal microvascular damage [29]. Blood pressure is closely linked to generalized endothelial dysfunction and subclinical atherosclerosis, which may also be another pathophysiological mechanism of blood pressure-related early symptoms of renal damage [30]. Therefore, blood pressure may be on the causal pathway between night shift work and decreased eGFR.

One key assumption that explains these associations is that night shift work induces misalignment between the external behavioral and the endogenous molecular circadian clocks. Kidneys are organs with peripheral circadian clocks, which enable GFR to show a self-sustained rhythmicity. Studies in animals and humans have shown that feeding time plays a dominant role in resetting peripheral circadian oscillators, even in the absence of the synchronization of the central circadian clock in the suprachiasmatic nucleus (SCN) [31,32]. Since light is the primary external world synchronizer of the central circadian clock in SCN, it seems logical to predict that chronic circadian disruption due to night shift work may result in decreased eGFR by influencing the peripheral oscillators. Therefore, altered meal timing due to night shift work (rather than LAN) could explain, at least in part, the relationship between night shift work and eGFR. In addition, the disruption of circadian sleep/wake rhythms are much more prevalent in end-stage renal disease, which indicates that the displaced sleep/wake cycle due to night shift work may also be responsible for the decline of GFR [33]. In terms of sleep, it is noteworthy that in addition to sleep rhythm (the sleep–wake cycle), sleep duration and sleep quality may also play a role in renal function [10,34,35]. Moreover, a link also exists between shift work and insufficient sleep [36]. However, the relationship between night shift work and eGFR was not modified or mediated by sleep duration and AIS score in our study, which suggests that the disruption of the sleep–wake cycle due to rotating night shift work may have a more pronounced effect on the circadian rhythm of GFR than sleep duration or sleep quality [7].

Another potential mechanism by which night shift work might cause the decline of eGFR is the presence of psychological and psychosocial stressors [11]. Shift-workers are subject to heavier stress loads compared to non-shift workers [37]. Stress can cause renal vasoconstriction by stimulating the sympathetic nervous system, resulting in decreased renal plasma flow (RPF) and GFR. Besides, persistent stimulation of the hypothalamic-pituitary-adrenal (HPA) axis by external chronic stressors due to night shift work can activate the sympathetic nervous system (SNS), and the activation of renal SNS may also affect the renal function through the renin-angiotensin-aldosterone system (RAAS) [12]. Moreover, the over-activation of RAAS not only leads to an increase in intra-glomerular pressure but also leads to the damage of vascular endothelial cells, the activation of reactive oxygen species (ROS), and the inhibition of sympathetic hyperactivity and nitric oxide (NO), which are known risk factors of renal damage [38,39].

The major strengths of our study include the detailed shift work information, lifestyle information, health status related to CKD, and large sample size. However, our research also has certain limitations. First, we are unable to infer temporality of shift work and GFR in a cross-sectional study. Second, the assessment of LAN is based on the self-reported information rather than the objective measurement of intensity. Third, we did not collect chronotype information, which may have led to a confounding bias. Fourth, those who are competent for long-duration night shift work are more likely to have better physical fitness (healthy worker effect) or have acclimated to night shift work, which may result in an underestimation of the association between the exposure and outcome. Fifth, our survey population consisted of steelworkers, the vast majority of whom are male workers in north China, which limits our ability to generalize these results to the general population.

## 5. Conclusions

Long-term night shift work, but not the brightness of bedroom ambient LAN, is associated with early stage of renal dysfunction in steelworkers. Since hypertension is one of the main markers and/or risk factors of CKD progression, and blood pressure may mediate the relationship between night shift work and decreased eGFR, control of blood pressure among shift-workers is particularly important for the prevention of CKD. Meanwhile, well-designed prospective research should be conducted with objective assessment of LAN exposure to explore the effect of LAN on renal function.

## Figures and Tables

**Table 1:** Basic characteristics according to eGFR status.

Variables	Overall	Non-Decreased eGFR	Decreased eGFR	
	(*n* = 6869)	(*n* = 5860)	(*n* = 1009)	*p* Value
Age (year), mean ± SD	44.2 ± 8.0	43.6 ± 8.1	47.9 ± 6.3	<0.001
Sex, n (%)				
Male	6283 (91.5)	5370 (91.6)	913 (90.5)	0.226
Female	586 (8.5)	490 (8.4)	96 (9.5)	
Ethnicity, n (%)				0.494
Han	6727 (97.9)	5736 (97.9)	991 (98.2)	
Others	142 (2.1)	124 (2.1)	18 (1.8)	
BMI (kg/m^2^), mean ± SD	25.2 ± 3.4	25.1 ± 3.4	25.7 ± 3.3	<0.001
Smoking status, n (%)				0.196
Never	2817 (41.0)	2409 (41.1)	408 (40.4)	
Ever	549 (8.0)	454 (7.6)	95 (9.4)	
Current	3503 (51.0)	2997 (51.1)	506 (50.2)	
Alcohol consumption, n (%)				0.233
Never	3936 (57.3)	3353 (57.2)	583 (57.8)	
Ever	393 (5.7)	325 (5.6)	68 (6.7)	
Current	2540 (37.0)	2182 (37.2)	358 (35.5)	
Education level, n (%)				<0.001
Primary or illiterate	86 (1.3)	71 (1.2)	15 (1.5)	
Middle or high school	5326 (77.5)	4483 (76.5)	843 (83.6)	
University or college	1457 (21.2)	1306 (22.3)	151 (15.0)	
Physical activity, n (%)				0.270
Low	80 (1.2)	70 (1.2)	10 (1.0)	
Moderate	522 (7.6)	457 (7.8)	65 (6.4)	
High	6267 (91.2)	5333 (91.0)	934 (92.6)	
Duration of night shift work (years), n (%)				<0.001
Day work	1029 (15.0)	896 (15.3)	133 (13.2)	
Q1 (1–12)	1471 (21.4)	1317 (22.5)	154 (15.3)	
Q2 (13–20)	1495 (21.8)	1328 (22.7)	167 (16.6)	
Q3 (21–28)	1314 (19.1)	1102 (18.8)	212 (21.0)	
Q4 (29–43)	1560 (22.7)	1217 (20.8)	343 (34.0)	
Brightness of bedroom ambient LAN, n (%)				0.856
Darkest level	3782 (55.1)	3222 (55.0)	560 (55.5)	
Middle level	2276 (33.1)	1949 (33.2)	327 (32.4)	
Lightest level	811 (11.8)	689 (11.8)	122 (12.1)	
Living duration of current residence, n (%)				0.899
<5 years	2191 (31.9)	1862 (31.8)	329 (32.6)	
6–10 years	1642 (23.9)	1400 (23.9)	242 (24.0)	
11–20 years	1682 (24.5)	1444 (24.6)	238 (23.4)	
>20 years	1354 (19.7)	1154 (19.7)	200 (19.8)	
Number of times light on (times/night), n (%)				0.645
0	4194 (61.1)	3584 (61.2)	610 (60.5)	
1	2172 (31.6)	1854 (31.6)	318 (31.5)	
≥2	503 (7.3)	422 (7.2)	81 (8.0)	
Sleep duration (h), mean ± SD	6.8 ± 1.2	6.8 ± 1.2	6.8 ± 1.2	0.005
Insomnia, n (%)				0.990
No	4560 (66.4)	3890 (66.4)	670 (66.4)	
Yes	2309 (33.6)	1970 (33.6)	339 (33.6)	
Diabetes, n (%)				0.150
No	6147 (89.5)	5257 (89.7)	890 (88.2)	
Yes	722 (10.5)	603 (10.3)	119 (11.8)	
Fasting blood glucose (mmol/L), median (IQR)	5.8 (5.4 to 6.3)	5.8 (5.4 to 6.3)	5.9 (5.5 to 6.4)	<0.001
Hypertension, n (%)				<0.001
No	5109 (74.4)	4447 (75.9)	662 (65.6)	
Yes	1760 (25.6)	1413 (24.1)	347 (34.4)	
Systolic blood pressure (mmHg), mean ± SD	129.0 ± 16.0	128.5 ± 15.8	131.8 ± 17.2	<0.001
Diastolic blood pressure (mmHg), mean ± SD	82.5 ± 10.4	82.2 ± 10.2	84.7 ± 11.0	<0.001
Hyperuricemia, n (%)				<0.001
No	4586 (66.8)	4070 (60.5)	516 (51.1)	
Yes	2283 (33.2)	1790 (30.6)	493 (48.9)	
Serum uric acid (μmol/L), mean ± SD	385.8 ± 94.2	385.3 ± 94.7	389.2 ± 91.1	0.244
^a^ Proteinuria (mg/dl), n (%)				<0.001
A1 (<30)	6745 (98.2)	5772 (98.5)	973 (96.4)	
A2-A3 (≥30)	124 (1.8)	88 (1.5)	36 (3.6)	

Values are expressed as the mean ± standard deviation (SD) or median (interquartile range (IQR)) or number (%); *p-*values were from Pearson’s chi-square test for categorical variables and Student’s t-test or Wilcoxon Scores (Rank Sums) for continuous variables. ^a^ Categories of proteinuria were defined as negative and trace (proteinuria < 30 mg/dl, A1), 1+ (proteinuria: 30–300 mg/dl, A2), and 2+ (proteinuria > 300 mg/dl, A3). SD, standard deviation; IQR, interquartile range; BMI, body mass index; eGFR, estimated glomerular filtration rate.

**Table 2:** Independent effect of night shift work and bedroom ambient light level on decreased eGFR.

Exposure Metrics	Decreased eGFR		OR (95% CI)		
	No, n (%)	Yes, n (%)	Model 1	Model 2	Model 3
Duration of night shift (years)					
Day work	896 (15.3)	133 (13.2)	1.00	1.00	1.00
Q1 (1–12)	1317 (22.5)	154 (15.3)	0.79 (0.61 to 1.01)	1.10 (0.85 to 1.42)	1.03 (0.79 to 1.34)
Q2 (13–20)	1328 (22.7)	167 (16.6)	0.84 (0.66 to 1.08)	1.12 (0.87 to 1.44)	1.01 (0.78 to 1.31)
Q3 (21–28)	1102 (18.8)	212 (21.0)	1.29 (1.02 to 1.63)	1.32 (1.04 to 1.68)	1.28 (1.01 to 1.64)
Q4 (29–43)	1217 (20.8)	343 (34.0)	1.89 (1.52 to 2.36)	1.40 (1.12 to 1.75)	1.37 (1.09 to 1.73)
*p* for trend			<0.001	<0.001	0.001
Brightness of bedroom ambient LAN					
Darkest level	3222 (55.0)	560 (55.5)	1.00	1.00	1.00
Middle level	1949 (33.3)	327 (32.4)	0.96 (0.83 to 1.12)	0.91 (0.78 to 1.06)	0.90 (0.77 to 1.05)
Lightest level	689 (11.8)	122 (12.1)	0.99 (0.80 to 1.23)	0.97 (0.78 to 1.21)	0.94 (0.75 to 1.18)

Values are expressed as number (%) or OR (95% CI). Model 1: unadjusted; Model 2: adjusted for age and sex; Model 3: further adjusted for BMI (<25, 25–30, or ≥30 kg/m^2^), smoking status, drinking status, education level, sleep duration (<7 or ≥7 h), living duration of current residence, number of times light on, insomnia, diabetes, hypertension, and hyperuricemia. OR, odds ratio; CI, confidence interval; BMI, body mass index; eGFR, estimated glomerular filtration rate.

**Table 3:** Associations between duration of night shifts and decreased eGFR stratified by sex, BMI, brightness of bedroom ambient LAN, diabetes, hypertension, hyperuricemia, insomnia, and short sleep duration.

Groups		Duration of Night Shifts (Years)				
	Day Work	Q1 (1–12)	Q2 (13–20)	Q3 (21–28)	Q4 (29–43)	*p* for Interaction
Sex						0.128
Male, OR (95% CI)	1.00	0.99 (0.74 to 1.31)	1.02 (0.78 to 1.35)	1.36 (1.04 to 1.76)	1.40 (1.12 to 1.79)	
Female, OR (95% CI)	1.00	0.88 (0.40 to 1.95)	0.65 (0.29 to 1.47)	0.65 (0.29 to 1.47)	0.79 (0.34 to 1.87)	
BMI						0.725
<25, OR (95% CI)	1.00	1.13 (0.77 to 1.67)	1.23 (0.84 to 1.80)	1.48 (1.02 to 2.13)	1.57 (1.11 to 2.20)	
≥25, OR (95% CI)	1.00	0.92 (0.64 to 1.32)	0.84 (0.59 to 1.19)	1.11 (0.79 to 1.55)	1.18 (0.85 to 1.63)	
Brightness of bedroom ambient LAN						0.905
Darkest level, OR (95% CI)	1.00	1.03 (0.71 to 1.49)	1.05 (0.74 to 1.51)	1.37 (0.97 to 1.94)	1.45 (1.04 to 2.03)	
Middle or Lightest level, OR (95% CI)	1.00	1.06 (0.73 to 1.54)	1.00 (0.68 to 1.47)	1.23 (0.86 to 1.76)	1.32 (0.95 to 1.83)	
Diabetes						0.724
No, OR (95% CI)	1.00	0.98 (0.7 to 1.30)	0.99 (0.76 to 1.30)	1.26 (0.97 to 1.63)	1.29 (1.01 to 1.66)	
Yes, OR (95% CI)	1.00	1.56 (0.66 to 3.69)	1.11 (0.44 to 2.82)	1.49 (0.64 to 3.47)	1.88 (0.91 to 3.90)	
Hypertension						0.683
No, OR (95% CI)	1.00	1.01 (0.74 to 1.38)	0.95 (0.69 to 1.29)	1.20 (0.89 to 1.62)	1.38 (1.03 to 1.84)	
Yes, OR (95% CI)	1.00	1.06 (0.65 to 1.72)	1.21 (0.76 to 1.94)	1.50 (0.96 to 2.32)	1.37 (0.91 to 2.06)	
Hyperuricemia						0.077
No, OR (95% CI)	1.00	0.98 (0.68 to 1.42)	1.28 (0.90 to 1.82)	1.37 (0.98 to 1.91)	1.56 (1.15 to 2.12)	
Yes, OR (95% CI)	1.00	1.02 (0.69 to 1.49)	0.78 (0.53 to 1.15)	1.16 (0.80 to 1.70)	1.13 (0.78 to 1.64)	
Insomnia						0.833
No, OR (95% CI)	1.00	0.99 (0.72 to 1.36)	0.96 (0.70 to 1.32)	1.21 (0.89 to 1.63)	1.38 (1.04 to 1.84)	
Yes, OR (95% CI)	1.00	1.11 (0.70 to 178)	1.10 (0.70 to 1.75)	1.41 (0.91 to 2.20)	1.42 (0.93 to 2.15)	
Short sleep duration						0.896
No, OR (95% CI)	1.00	1.16 (0.77 to 1.74)	1.15 (0.77 to 1.71)	1.50 (1.03 to 2.19)	1.49 (1.05 to 2.12)	
Yes, OR (95% CI)	1.00	0.98 (0.69 to 1.38)	0.92 (0.65 to 1.30)	1.13 (0.81 to 1.59)	1.31 (0.95 to 1.81)	

*p-*values for interaction were estimated using a log likelihood ratio test to compare models with and without cross-product interaction terms. Adjusted for age, sex, BMI (<25, 25–30, or ≥30 kg/m^2^), smoking status, drinking status, education level, short sleep duration (<7 or ≥7 h), living duration of current residence, number of times light on, insomnia, diabetes, hypertension, and hyperuricemia (except for the stratification variable in each subgroup). OR, odds ratio; CI, confidence interval; BMI, body mass index; eGFR, estimated glomerular filtration rate.

**Table 4:** Mediation analysis of potential mediators on the association between duration of night shifts and eGFR.

Mediators	Effect Size (95% CI)	*p*-Value
Systolic blood pressure (mmHg)		
ACME	−0.021 (−0.060 to 0.006)	0.160
ADE	−1.188 (−1.916 to −0.458)	<0.001
Total effect	−1.210 (−1.924 to −0.483)	<0.001
Proportion of mediation	0.018 (−0.006 to 0.073)	0.160
Diastolic blood pressure (mmHg)		
ACME	−0.077 (−0.134 to −0.030)	<0.001
ADE	−1.133 (−1.851 to −0.409)	<0.001
Total effect	−1.210 (−1.925 to −0.483)	<0.001
Proportion of mediation	0.064 (0.024 to 0.167)	<0.001
Fasting blood glucose (mmol/L)		
ACME	0.037 (−0.021 to 0.093)	0.200
ADE	−1.231 (−1.919 to −0.504)	<0.001
Total effect	−1.194 (−1.885 to −0.463)	<0.001
Proportion of mediation	−0.031 (−0.113 to 0.018)	<0.001
Serum uric acid (μmol/L)		
ACME	−0.002 (−0.019 to 0.010)	0.730
ADE	−1.286 (−2.068 to −0.545)	0.004
Total effect	−1.288 (−2.070 to −0.548)	0.004
Proportion of mediation	0.002 (−0.009 to 0.015)	0.730
Sleep duration (hours)		
ACME	0.031 (−0.049 to 0.112)	0.480
ADE	−1.243 (−1.893 to −0.503)	<0.001
Total effect	−1.213 (−1.867 to −0.485)	<0.001
Proportion of mediation	−0.025 (−0.125 to 0.045)	<0.001
AIS score		
ACME	−0.014 (−0.047 to 0.005)	0.210
ADE	−1.200 (−1.882 to −0.466)	<0.001
Total effect	−1.214 (−1.896 to −0.489)	<0.001
Proportion of mediation	0.012 (−0.006 to 0.053)	0.210

Adjusted for age, sex, BMI (<25, 25–30, or ≥30 kg/m2), smoking status, drinking status, education level, short sleep duration (<7 or ≥7 h), living duration of current residence, number of times light on, insomnia, diabetes, hypertension, and hyperuricemia (mediator was included as continuous variable in each mediation analysis). BMI, body mass index; eGFR, estimated glomerular filtration rate; AIS, Athens Insomnia Scale; ACME, average causal mediation effect; ADE, average direct effect.

## Supplementary Materials

The following are available online at https://www.mdpi.com/1660-4601/17/23/9035/s1, Table S1: Basic characteristics of participants according to shift work status, Table S2: Basic characteristics of participants according to sex, Table S3: Independent effect of cumulative number of night shifts and bedroom ambient light level on decreased eGFR, Table S4: Independent effect of duration of night shifts and bedroom ambient light level on decreased eGFR after further adjustment for the main occupational hazards, Table S5: Independent effect of duration of night shifts and bedroom ambient light level on decreased eGFR after further adjustment for the previous history, medication status, and duration of diabetes, hypertension and glomerulonephritis as well as family history of CKD. Table S6: Independent effect of cumulative number of night shifts and bedroom ambient light level on decreased eGFR after further adjustment for the previous history, medication status and duration of diabetes, hypertension and glomerulonephritis as well as family history of CKD. Figure S1: Flow chart of selection of participants, Figure S2: Mediation analysis of potential mediators on the association between night shift work and eGFR, Figure S3: Associations of duration of night shifts (continuous), and cumulative number of night shifts (continuous) with eGFR from restricted cubic spline models after deleting the last 1% quantile of the duration of night shifts, cumulative number of night shifts.
