# Supplementary material for: Rotating Night Shift Work, Exposure to Light at Night, and Glomerular Filtration Rate: Baseline Results from a Chinese Occupational Cohort
Source: Int J Environ Res Public Health. 2020 Dec 4;17(23):9035. doi: 10.3390/ijerph17239035 (PMC7730862; doi:10.3390/ijerph17239035)
Supplement: Supplementary file 1 [file ijerph-17-09035-s001.pdf]

Supplementary material：

Rotating night shift work, exposure to light at night, and glomerular filtration rate: baseline results from a Chinese occupational cohort

Shengkui Zhang [1], Yongbin Wang [2], Ying Zhu [1], Xiaoming Li [1], Yang Song [1], Juxiang Yuan [1,*]

[1] Department of Epidemiology and Health Statistics, School of Public Health, North China University of Science and Technology, Tangshan, Hebei Province, China; zhangsk@stu.ncst.edu.cn (S.Z.); zhuying@ncst.edu.cn (Y.Z.); lixiaoming@ncst.edu.cn (X.L.); songyang@ncst.edu.cn (Y.S.); gwxyjxb@ncst.edu.cn (J.Y.)

[2] Department of Epidemiology and Health Statistics, School of Public Health, Xinxiang Medical University, Xinxiang, Henan Province, China; 191035@xxmu.edu.cn

* Correspondence: gwxyjxb@ncst.edu.cn; Tel.: +86-0315-8805578

Assessment of covariates

A structured questionnaire was used after repeated revisions. All information in the questionnaire was collected through face-to-face surveys. After checking the completeness and correctness of each questionnaire, we used a customized program to scan and transformed the handwritten data into an electronic data set immediately. The transformed version of each questionnaire was checked by two skilled investigators separately, and then the checked results were compared and reviewed before the final submission. The questionnaire mainly includes age, gender, ethnicity, work schedule, smoking, drinking, educational level, physical activity, sleep duration and insomnia. Smoking and drinking status were evaluated from self-reported information and were divided into "never", "ever" and "current". The level of education was divided into three categories: "primary or illiterate," "middle or high school," and "university or college." The calculation of metabolic equivalents was based on the International Physical Activity Questionnaire (IPAQ) [1]. The assessment of insomnia was estimated using the Athens Insomnia Scale (AIS), and AIS score ≥6 was defined as insomnia [2]. The duration of sleep were the weighted average value of sleep duration on working days and rest days, and <7 hours of sleep was defined as short sleep duration [3].

Standard study protocols were used to train qualified physicians and nurses prior to

this survey. Height and weight were measured three times each. The participants stood upright and barefoot in light clothes. The height and weight data that were ultimately used for analysis were accurate to 0.1 cm and 0.1 kg. Body mass index (BMI) was defined as body weight (kg) divided by the square of the body height (m^2). Blood pressure measurements were performed three times at five-minute intervals using an electronic sphygmomanometer (OMRON, HBP-1100, China), and the participants were required to rest for more than ten minutes. Finally, the mean was obtained for analysis. Elevated blood pressure was defined as current systolic blood pressure ≥140 mmHg, diastolic blood pressure ≥90 mmHg, or if the patient was receiving antihypertensive therapy. Participants were required to fast overnight before the abdominal ultrasound examination and blood collection. Participants' anterior elbow vein blood was collected and centrifuged at room temperature (3000 r/min, 15 minutes) immediately. All blood samples were tested in the central laboratory of Tangshan Hongci Hospital Laboratory using automatic biochemical analysers (mindrary, BS-800, China) within four hours. Serum uric acid concentrations greater than 6 mg/dL (357 μmol/L) for females, 7 mg/dL (416 μmol/L) for males, are defined as hyperuricemia [4].

All related occupational hazard factors were measured by a qualified third-party company in accordance with the National Occupational Health Standards of the People's Republic of China (ICS 13.100). Exposure to dust was defined as workers who may be exposed to productive dust (inorganic dust, organic dust or mixed dust) during production (GBZ/T 229.1–2010) [5]. The total dust in the air of the workplace was collected at the breathing zone with a filter membrane, and its concentration was calculated based on the increased weight of the filter membrane and the amount of gas collected. When the dust concentration in the air was ≤50 mg/m^3, a filter membrane with a diameter of 37 mm or 40 mm was used; otherwise, a filter membrane with a diameter of 75 mm was used (GBZ/T 192.1–2007) [6]. Exposure to heat stress work was defined as the average wet-bulb globe temperature (WBGT) index of the workplace being equal to or greater than 25°C in the process of production (GBZ 2.2–2007) [7]. The WBGT index was measured by a black-wet bulb globe thermometer. If there was no productive heat source in the workplace, three measuring points were selected to take the average value of the WBGT index; if where

there was a productive heat source, 3 to 5 measuring points were selected to take the average value of the WBGT index. If the workplace was isolated into different thermal or ventilated environments, 2 measuring points were selected to take the average value of the WBGT index (GBZ/T 189.7–2007) [8]. Exposure to industrial toxicants was defined as workers who may be exposed to a variety of harmful chemicals (the toxicant specifically refers to carbon monoxide in this population) during production (GBZ/T 229.2–2010) [9]. Carbon monoxide or carbon dioxide in the air of the workplace was pumped into a non-dispersive infrared-ray (NDIR) analyzer and selectively absorbed its infrared rays. The concentration of carbon monoxide was determined according to the absorption value (GBZ/T 160.28–2004) [10]. Exposure to noise was defined as workers who were exposed to a noisy environment where the 8-h/d or 40-h/week equivalent A-weighted sound pressure level was ≥80 dB, which may be harmful to health and hearing (GBZ/T 229.4–2012) [11]. The workplace production noise was measured by a sound level meter. If the distribution of the sound field in the workplace was uniform (between-field difference of A-sound levels were less than 3 dB(A)), three measuring points were selected to take the average value; otherwise, the workplace was divided into several sound level areas. In each sound field, two measuring points were selected to take the average value (GBZ/T 189.8–2007) [12].

Table of contents

Table S1 Basic characteristics of participants according to shift work status

Table S2 Basic characteristics of participants according to sex

Table S3 Independent effect of cumulative number of night shifts and bedroom ambient light level on decreased eGFR

Table S4 Independent effect of duration of night shifts and bedroom ambient light level on decreased eGFR after further adjustment for the main occupational hazards

Table S5 Independent effect of duration of night shifts and bedroom ambient light level on decreased eGFR after further adjustment for the previous history, medication status,

and duration of diabetes, hypertension and glomerulonephritis as well as family history of CKD

Table S6 Independent effect of cumulative number of night shifts and bedroom ambient light level on decreased eGFR after further adjustment for the previous history, medication status and duration of diabetes, hypertension and glomerulonephritis as well as family history of CKD

Figure of contents

Figure S1 Flow chart of selection of participants.

Figure S2 Mediation analysis of potential mediators on the association between night shift work and eGFR.

Figure S3 Associations of duration of night shifts (continuous), and cumulative number of night shifts (continuous) with eGFR from restricted cubic spline models after deleting the last 1% quantile of the duration of night shifts, cumulative number of night shifts.

Table S1 Basic characteristics of participants according to shift work status

Variables	Overall	Shift work status		
		Day work	Night shift work	
	(*N*=6869)	(n=1029)	(n=5840)	*P* value
Age (year), mean ± SD	44.2 ± 8.0	44.4 ± 9.1	44.2 ± 7.8	0.558
Sex, n (%)				
Male	6283 (91.5)	910 (88.4)	5373 (92.0)	<0.001
Female	586 (8.5)	119 (11.6)	467 (8.0)	
Ethnicity, n (%)				0.571
Han	6727 (97.9)	1005 (97.7)	5722 (98.0)	
Other	142 (2.1)	24 (2.3)	118 (2.0)	
BMI (kg/m^2), n (%)	25.2 ± 3.4	24.8 ± 3.3	25.3 ± 3.4	<0.001
Smoking status, n (%)				<0.001
Never	2817 (41.0)	461 (44.8)	2356 (40.3)	
Ever	549 (8.0)	53 (5.2)	496 (8.5)	
Current	3503 (51.0)	515 (50.1)	2988 (51.2)	
Alcohol consumption, n (%)				<0.001
Never	3936 (57.3)	632 (61.4)	3304 (56.6)	
Ever	393 (5.7)	26 (2.5)	367 (6.3)	
Current	2540 (37.0)	371 (36.1)	2169 (37.1)	
Education level, n (%)				<0.001
Primary or illiterate	86 (1.3)	9 (0.9)	77 (1.3)	
Middle or high school	5326 (77.5)	712 (69.2)	4614 (79.0)	
University or college	1457 (21.2)	308 (29.9)	1149 (19.7)	
Physical activity, n (%)				0.067
Low	80 (1.2)	6 (0.6)	74 (1.3)	
Moderate	522 (7.6)	68 (6.6)	454 (7.8)	
High	6267 (91.2)	955 (92.8)	5312 (91.0)	
Bedroom ambient light level, n (%)				<0.001
Darkest level	3782 (55.1)	484 (47.0)	3298 (56.5)	
Middle level	2276 (33.1)	412 (40.0)	1864 (31.9)	
Lightest level	811 (11.8)	133 (12.9)	678 (11.6)	
Number of times light on (times/night), n (%)				
0	4194 (61.1)	674 (65.5)	923 (61.5)	0.018
1	2172 (31.6)	284 (27.6)	467 (31.3)	
≥2	503 (7.3)	71 (6.9)	110 (7.3)	
Sleep duration (h), mean ± SD	6.8 ± 1.2	7.0 ± 1.2	6.7 ± 1.2	<0.001
Insomnia, n (%)				0.064
No	4560 (66.4)	709 (68.9)	3851 (65.9)	
Yes	2309 (33.6)	320 (31.1)	1989 (34.1)	
Diabetes, n (%)				0.497
No	6147 (89.5)	927 (90.1)	5220 (89.4)	
Yes	722 (10.5)	102 (9.9)	620 (10.6)	

Fasting blood glucose (mmol/L), median (IQR)	5.8 (5.4 to 6.3)	5.8 (5.4 to 6.3)	5.8 (5.4 to 6.3)	0.868
Hypertension, n (%)				0.503
No	5109 (74.4)	774 (75.2)	4335 (74.2)	
Yes	1760 (25.6)	255 (24.8)	1505 (25.8)	
Systolic blood pressure (mmHg), mean ± SD	129.0 ± 16.0	127.5 ± 16.1	129.2 ± 16.0	0.002
Diastolic blood pressure (mmHg), mean ± SD	82.5 ± 10.4	81.3 ± 10.3	82.8 ± 10.4	<0.001
Hyperuricemia, n (%)				0.003
No	4586 (66.8)	729 (70.9)	3857 (66.0)	
Yes	2283 (33.2)	300 (29.2)	1983 (34.0)	
Serum uric acid (μmol/L), mean ± SD	385.8 ± 94.2	380.8 ± 94.9	386.7 ± 94.0	0.076
Range of eGFR, n (%)				0.100
G1 (≥90)	5860 (85.3)	896 (87.1)	4964 (85.0)	
G2 (60–89)	987 (14.4)	128 (12.4)	859 (14.7)	
G3a-G5 (<60)	22 (0.3)	5 (0.5)	17 (0.3)	
eGFR (mL/min/1.73 m^2), mean ± SD	101.7 ± 11.4	102.3 ± 11.4	101.6 ± 11.4	0.059
Proteinuria (mg/dl), n (%)				0.157
A1 (<30)	6745 (98.2)	1016 (98.7)	5729 (98.1)	
A2-A3 (≥30)	124 (1.8)	13 (1.3)	111 (1.9)	

Values are expressed as the mean ± SD or median (IQR) or number (%); *P*-values were from Pearson's chi-square test for categorical variables and Student's t test or Wilcoxon Scores (Rank Sums) for continuous variables. IQR, interquartile range; BMI, body mass index; eGFR, estimated glomerular filtration rate.

Table S2 Basic characteristics of participants according to sex

Variables	Overall	Female	Male	
	(*N*=6869)	(n=586)	(n=6283)	*P* value
Age (year), mean ± SD	44.2 ± 8.0	44.1 ± 5.1	44.3 ± 8.3	0.391
Ethnicity				0.212
Han	6727 (97.9)	578 (98.6)	6149 (97.9)	
Other	142 (2.1)	8 (1.4)	134 (2.1)	
BMI (kg/m^2), n (%)	25.2 ± 3.4	23.7 ± 3.1	25.3 ± 3.4	<0.001
Smoking status, n (%)				<0.001
Never	2817 (41.0)	514 (87.7)	2303 (36.7)	
Ever	549 (8.0)	20 (3.4)	529 (8.4)	
Current	3503 (51.0)	52 (8.9)	3451 (54.9)	
Alcohol consumption, n (%)				<0.001
Never	3936 (57.3)	527 (89.9)	3409 (54.3)	
Ever	393 (5.7)	23 (3.9)	370 (5.9)	
Current	2540 (37.0)	36 (6.1)	2504 (39.9)	
Education level, n (%)				0.657
Primary or illiterate	86 (1.3)	5 (0.9)	81 (1.3)	
Middle or high school	5326 (77.5)	455 (77.7)	4871 (77.5)	
University or college	1457 (21.2)	126 (21.5)	1331 (21.2)	
Physical activity, n (%)				0.736

Low	80 (1.2)	5 (0.9)	75 (1.2)	
Moderate	522 (7.6)	43 (7.3)	479 (7.6)	
High	6267 (91.2)	538 (91.8)	5729 (91.2)	
Duration of night shift (years), n (%)				
Day work	1029 (15.0)	119 (20.3)	910 (14.5)	
Q1 (1–12)	1471 (21.4)	122 (20.8)	1349 (21.5)	
Q2 (13–20)	1495 (21.8)	130 (22.2)	1365 (21.7)	
Q3 (21–28)	1314 (19.1)	129 (22.0)	1185 (18.9)	
Q4 (29–43)	1560 (22.7)	86 (14.7)	1474 (23.5)	
Bedroom ambient light level, n (%)				<0.001
Darkest level	3782 (55.1)	265 (45.2)	3517 (56.0)	
Middle level	2276 (33.1)	265 (45.2)	2011 (32.0)	
Lightest level	811 (11.8)	56 (9.6)	755 (12.0)	
Number of times light on (times/night), n (%)				0.271
0	4194 (61.1)	376 (64.2)	3818 (60.8)	
1	2172 (31.6)	170 (29.0)	2002 (31.9)	
≥2	503 (7.3)	40 (6.8)	463 (7.4)	
Sleep duration (h), mean ± SD	6.8 ± 1.2	6.7 ± 1.2	6.8 ± 1.2	0.328
Insomnia, n (%)				0.785
No	4560 (66.4)	392 (66.9)	4168 (66.3)	
Yes	2309 (33.6)	194 (33.1)	2115 (33.7)	
Diabetes, n (%)				<0.001
No	6147 (89.5)	555 (94.7)	5592 (89.0)	
Yes	722 (10.5)	31 (5.3)	691 (11.0)	
Fasting blood glucose (mmol/L), median (IQR)	5.8 (5.4 to 6.3)	5.6 (5.3 to 5.9)	5.8 (5.5 to 6.3)	<0.001
Hypertension, n (%)				<0.001
No	5109 (74.4)	514 (87.7)	4595 (73.1)	
Yes	1760 (25.6)	72 (12.3)	1688 (26.9)	
Systolic blood pressure (mmHg), mean ± SD	129.0 ± 16.0	122.6 ± 14.8	129.5 ± 16.0	<0.001
Diastolic blood pressure (mmHg), mean ± SD	82.5 ± 10.4	78.0 ± 9.7	83.0 ± 10.3	<0.001
Hyperuricemia, n (%)				<0.001
No	4586 (66.8)	480 (81.9)	4106 (65.4)	
Yes	2283 (33.2)	106 (18.1)	2177 (34.7)	
Serum uric acid (μmol/L), mean ± SD	385.8 ± 94.2	383.1 ± 93.5	386.1 ± 94.3	0.485
Range of eGFR, n (%)				0.376
G1 (≥90)	5860 (85.3)	490 (83.6)	5370 (85.5)	
G2 (60–89)	987 (14.4)	93 (15.9)	894 (14.2)	
G3a-G5 (<60)	22 (0.3)	3 (0.5)	19 (0.3)	
eGFR (mL/min/1.73 m^2), mean ± SD	101.7 ± 11.4	102.0 ± 13.0	101.7 ± 11.3	0.490
Proteinuria (mg/dl), n (%)				0.609
A1 (<30)	6745 (98.2)	577 (98.5)	6168 (98.2)	
A2-A3 (≥30)	124 (1.8)	9 (1.5)	115 (1.8)	

Values are expressed as the mean ± SD or median (IQR) or number (%); *P*-values were from Pearson's chi-square test for categorical variables and Student's t test or Wilcoxon Scores (Rank Sums) for continuous variables. IQR, interquartile range; BMI, body mass index; eGFR, estimated glomerular filtration rate.

Table S3 Independent effect of cumulative number of night shifts and bedroom ambient light level on decreased eGFR

Exposure metrics	Decreased eGFR		OR (95% CI)		
	No, n (%)	Yes, n (%)	Model 1	Model 2	Model 3
Cumulative number of night shifts (nights)					
Day work	896 (15.3)	133 (13.2)	1.00	1.00	1.00
Q1 (43–1132)	1311 (22.4)	149 (14.8)	0.76 (0.60 to 0.98)	1.07 (0.83 to 1.39)	1.01 (0.77 to 1.31)
Q2 (1133–1854)	1297 (22.1)	163 (16.2)	0.84 (0.66 to 1.08)	1.13 (0.88 to 1.46)	1.03 (0.79 to 1.33)
Q3 (1855–2584)	1213 (20.7)	248 (24.6)	1.37 (1.09 to 1.72)	1.40 (1.11 to 1.76)	1.36 (1.07 to 1.74)
Q4 (2585–5239)	1143 (19.5)	316 (31.3)	1.86 (1.49 to 2.32)	1.35 (1.07 to 1.69)	1.31 (1.04 to 1.66)
P for trend			<0.001	0.001	0.001
Brightness of bedroom ambient LAN					
Darkest level	3222 (55.0)	560 (55.5)	1.00	1.00	1.00
Middle level	1949 (33.3)	327 (32.4)	0.96 (0.83 to 1.12)	0.92 (0.79 to 1.07)	0.90 (0.77 to 1.06)
Lightest level	689 (11.8)	122 (12.1)	1.00 (0.80 to 1.24)	0.98 (0.79 to 1.21)	0.95 (0.76 to 1.19)

Model 1: unadjusted; Model 2: adjusted for age and gender; Model 3: further adjusted for BMI (<25, 25–30, or ≥30 kg/m^2), smoking status, drinking status, education level, sleep duration (<7, or ≥7 hours), living duration of current residence, number of times light on, insomnia, diabetes, hypertension, and hyperuricemia.

Table S4 Independent effect of duration of night shifts and bedroom ambient light level on decreased eGFR after further adjustment for the main occupational hazards

Exposure metrics	Decreased eGFR		OR (95% CI)
	No, n (%)	Yes, n (%)	
Duration of night shift (years)			
Day work	896 (15.3)	133 (13.2)	1.00
Q1 (1–12)	1317 (22.5)	154 (15.3)	1.02 (0.78 to 1.32)
Q2 (13–20)	1328 (22.7)	167 (16.6)	0.99 (0.76 to 1.29)
Q3 (21–28)	1102 (18.8)	212 (21.0)	1.26 (0.98 to 1.62)
Q4 (29–43)	1217 (20.8)	343 (34.0)	1.35 (1.07 to 1.72)
P for trend			0.002
Bedroom ambient light level			
Darkest level	3222 (55.0)	560 (55.5)	1.00
Middle level	1949 (33.3)	327 (32.4)	0.89 (0.76 to 1.04)
Lightest level	689 (11.8)	122 (12.1)	0.94 (0.75 to 1.18)

Adjusted for age, gender, BMI (<25, 25–30, or ≥30 kg/m^2), smoking status, drinking status, education level, sleep duration (<7, or ≥7 hours), living duration of current residence, number of times light on, insomnia, diabetes, hypertension, hyperuricemia, dust exposure (No or Yes), heat stress exposure (No or Yes), noise exposure (No or Yes), and carbon monoxide exposure (No or Yes).

Table S5 Independent effect of duration of night shifts and bedroom ambient light level on decreased eGFR after further adjustment for the previous history, medication status, and duration of diabetes, hypertension and glomerulonephritis as well as family history of CKD

Exposure metrics	Decreased eGFR		OR (95% CI)
	No, n (%)	Yes, n (%)	
Duration of night shift (years)			
Day work	896 (15.3)	133 (13.2)	1.00
Q1 (1–12)	1317 (22.5)	154 (15.3)	1.03 (0.80 to 1.34)
Q2 (13–20)	1328 (22.7)	167 (16.6)	1.01 (0.78 to 1.31)
Q3 (21–28)	1102 (18.8)	212 (21.0)	1.28 (0.99 to 1.63)
Q4 (29–43)	1217 (20.8)	343 (34.0)	1.36 (1.08 to 1.72)
P for trend			0.001
Bedroom ambient light level			
Darkest level	3222 (55.0)	560 (55.5)	1.00
Middle level	1949 (33.3)	327 (32.4)	0.90 (0.77 to 1.05)
Lightest level	689 (11.8)	122 (12.1)	0.95 (0.76 to 1.19)

Adjusted for age, gender, BMI (<25, 25–30, or ≥30 kg/m^2), smoking status, drinking status, education level, sleep duration (<7, or ≥7 hours), living duration of current residence, number of times light on, insomnia, diabetes, hypertension, hyperuricemia, previous history of diabetes (No or Yes), hypertension (No or Yes) and glomerulonephritis (No or Yes), duration of diabetes (years), hypertension (years) and glomerulonephritis (years), whether to take drugs for diabetes (No or Yes), hypertension (No or Yes) and glomerulonephritis (No or Yes) in the past two weeks, and family history of CKD (No or Yes).

Table S6 Independent effect of cumulative number of night shifts and bedroom ambient light level on decreased eGFR after further adjustment for the previous history, medication status and duration of diabetes, hypertension and glomerulonephritis as well as family history of CKD

Exposure metrics	Decreased eGFR		OR (95% CI)
	No, n (%)	Yes, n (%)	
Cumulative number of night shifts (nights)			
Day work	896 (15.3)	133 (13.2)	1.00
Q1 (43–1132)	1311 (22.4)	149 (14.8)	1.01 (0.77 to 1.31)
Q2 (1133–1854)	1297 (22.1)	163 (16.2)	1.02 (0.79 to 1.33)
Q3 (1855–2584)	1213 (20.7)	248 (24.6)	1.36 (1.07 to 1.73)

Q4 (2585–5239)	1143 (19.5)	316 (31.3)	1.31 (1.03 to 1.65)
P for trend			0.002
Brightness of bedroom ambient LAN			
Darkest level	3222 (55.0)	560 (55.5)	1.00
Middle level	1949 (33.3)	327 (32.4)	0.90 (0.77 to 1.06)
Lightest level	689 (11.8)	122 (12.1)	0.96 (0.76 to 1.20)

Adjusted for age, gender, BMI (<25, 25–30, or ≥30 kg/m^2), smoking status, drinking status, education level, sleep duration (<7, or ≥7 hours), living duration of current residence, number of times light on, insomnia, diabetes, hypertension, hyperuricemia, previous history of diabetes (No or Yes), hypertension (No or Yes) and glomerulonephritis (No or Yes), duration of diabetes (years), hypertension (years) and glomerulonephritis (years), whether to take drugs for diabetes (No or Yes), hypertension (No or Yes) and glomerulonephritis (No or Yes) in the past two weeks, and family history of CKD (No or Yes).

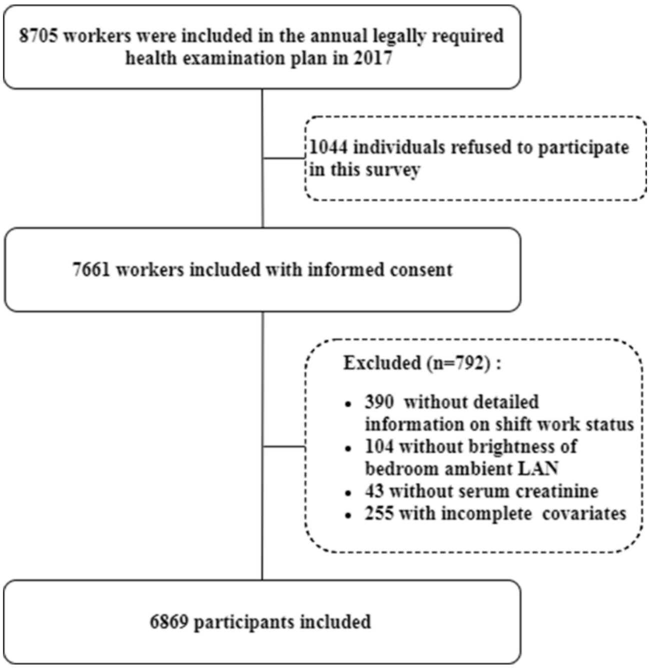

Figure S1 Flow chart of selection of participants.

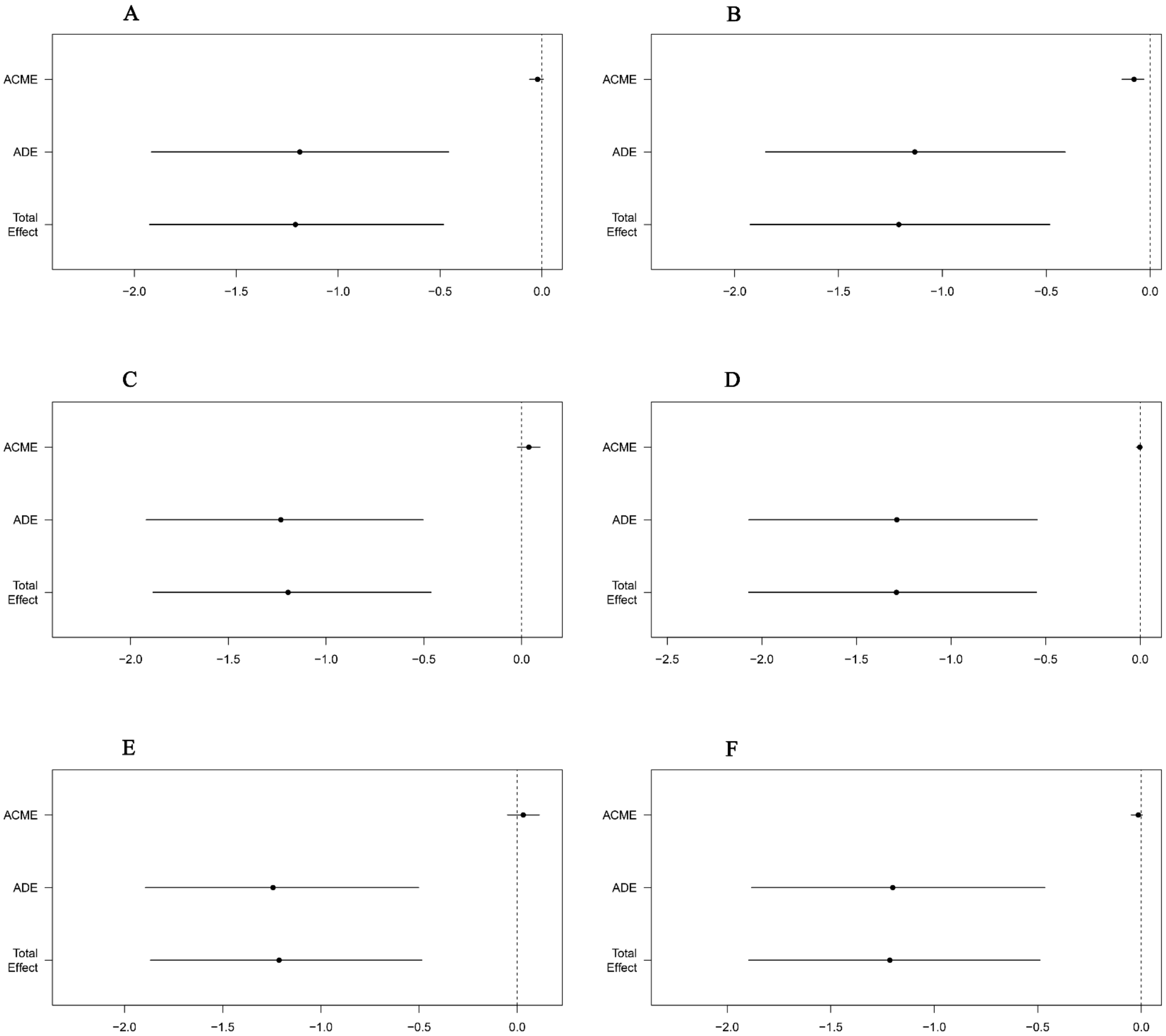

Figure S2 Mediation analysis of potential mediators on the association between night shift work and eGFR. A, Systolic blood pressure (mmHg); B, Diastolic blood pressure (mmHg); C, Fasting blood glucose (mmol/L); D, Serum uric acid (μmol/L); E, Sleep duration (hours); F, AIS score. Adjusted for age, sex, BMI (<25, 25–30, or ≥30 kg/m^2), smoking status, drinking status, education level, short sleep duration (<7, or ≥7 hours), living duration of current residence, number of times light on, insomnia, diabetes, hypertension, and hyperuricemia (mediator was included as continuous variable in each mediation analysis). BMI, body mass index; eGFR, estimated glomerular filtration rate; AIS, Athens Insomnia Scale; ACME, average causal mediation effect; ADE, average direct effect.

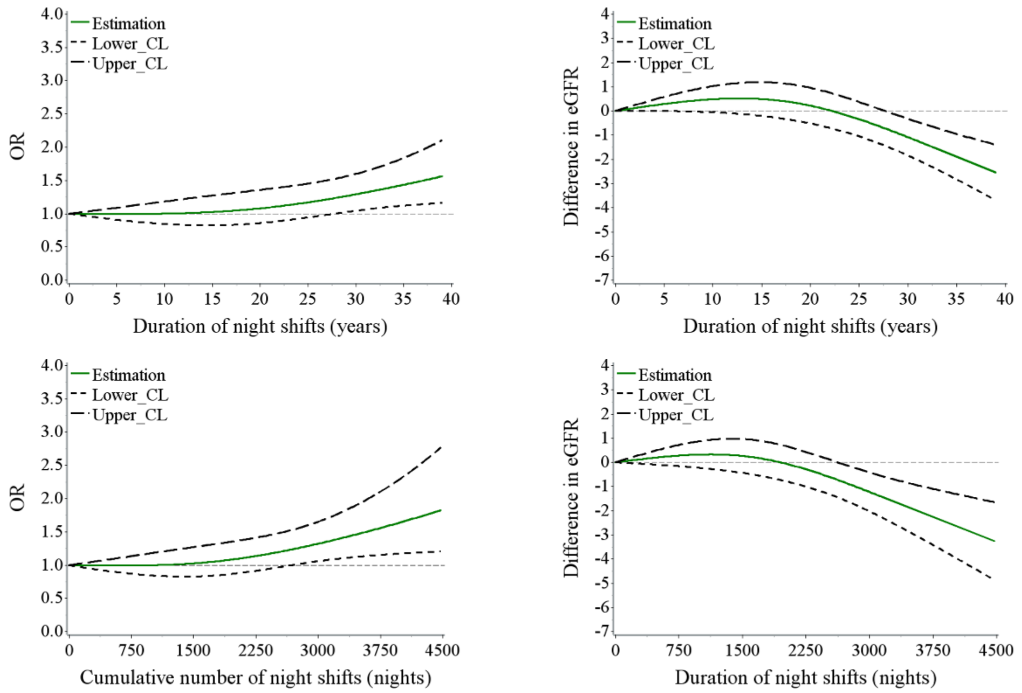

Figure S3 Associations of duration of night shift work (continuous), and cumulative number of night shifts (continuous) with eGFR from restricted cubic spline models after deleting the last 1% quantile of the duration of night shifts, cumulative number of night shifts. "Difference in eGFR" indicates difference in eGFR (mL/min/1.73 m^2) where the reference values for duration of night shifts and cumulative number of night shifts are all 0 (day work). Adjusted for age, sex, BMI (<25, 25–30, or ≥30 kg/m^2), smoking status, drinking status, education level, short sleep duration (<7, or ≥7 hours), living duration of current residence, number of times light on, insomnia, diabetes, hypertension, and hyperuricemia. BMI, body mass index; eGFR, estimated glomerular filtration rate; OR, odds ratio; CI, 95% confidence interval.

REFERENCES

1. Celis-Morales, C. A.; Perez-Bravo, F.; Ibanez, L.; Salas, C.; Bailey, M. E.; Gill, J. M., Objective vs. self-reported physical activity and sedentary time: effects of measurement method on relationships with risk biomarkers. *PLoS One* **2012,** 7, (5), e36345.
2. Soldatos, C. R.; Dikeos, D. G.; Paparrigopoulos, T. J., Athens Insomnia Scale: validation of an instrument based on ICD-10 criteria. *J. Psychosom. Res.* **2000,** 48, (6), 555-60.
3. Shockey, T. M.; Wheaton, A. G., Short Sleep Duration by Occupation Group - 29 States, 2013-2014. *MMWR. Morbidity and mortality weekly report* **2017,** 66, (8), 207-213.
4. Gois, P. H. F.; Souza, E. R. M., Pharmacotherapy for hyperuricaemia in hypertensive patients. *The Cochrane database of systematic reviews* **2020,** 9, Cd008652.

5. GBZ/T GBZ/T 229.1–2010 Classification of occupational hazards at workplaces. Part 1: Occupational exposure to industrial dust. http://niohp.chinacdc.cn/zyysjk/zywsbzml/201210/t20121012_70490.htm
6. GBZ/T 192.1–2007 Determination of dust in the air of workplace. Part 1: Total dust concentration. http://niohp.chinacdc.cn/zyysjk/zywsbzml/201210/t20121012_70522.htm
7. GBZ 2.2–2007 Occupational exposure limits for hazardous agents in the workplace. Part 2: Physical agents. http://niohp.chinacdc.cn/zyysjk/zywsbzml/201303/t20130329_79199.htm
8. GBZ/T 189.7–2007 Measurement of physical agents in workplace. Part 7: Heat Stress. http://niohp.chinacdc.cn/zyysjk/zywsbzml/201210/t20121012_70527.htm
9. GBZ/T 229.2–2010 Classification of occupational hazards at workplaces. Part 2: Occupational exposure to chemicals. http://niohp.chinacdc.cn/zyysjk/zywsbzml/201210/t20121012_70489.htm
10. GBZ/T 160.28–2004 Methods for determination of inorganic carbon compounds in the air of workplace. http://niohp.chinacdc.cn/zyysjk/zywsbzml/201210/t20121015_70624.htm
11. GBZ/T 229.4–2012 Classification of occupational hazards at workplaces. Part 4: Occupational exposure to noise. http://niohp.chinacdc.cn/zyysjk/zywsbzml/201307/t20130715_84934.htm
12. GBZ/T 189.8–2007 Measurement of physical agents in workplace. Part 8: Noise. http://niohp.chinacdc.cn/zyysjk/zywsbzml/201210/t20121012_70526.htm
